# Relationships between medical students’ co-regulatory network characteristics and self-regulated learning: a social network study

**DOI:** 10.1007/s40037-021-00664-x

**Published:** 2021-04-30

**Authors:** Derk Bransen, Marjan J. B. Govaerts, Dominique M. A. Sluijsmans, Jeroen Donkers, Piet G. C. Van den Bossche, Erik W. Driessen

**Affiliations:** 1 School of Health Professions Education (SHE), Maastricht University, Maastricht, The Netherlands; 2 Department of Educational Development and Research, Faculty of Health, Medicine and Life Science, Maastricht University, Maastricht, The Netherlands; 3 Faculty of Social Sciences, Radboud University, Nijmegen, The Netherlands; 4 Department of Training and Education, Faculty of Social Sciences, University of Antwerp, Antwerp, Belgium; 5 Department of Educational Research and Development, School of Business and Economics, Maastricht University, Maastricht, The Netherlands

**Keywords:** Self-regulated learning, Co-regulated learning, Network characteristics, Social network analysis, Clinical clerkship contexts

## Abstract

**Introduction:**

Recent conceptualizations of self-regulated learning acknowledge the importance of co-regulation, i.e., students’ interactions with others in their networks to support self-regulation. Using a social network approach, the aim of this study is to explore relationships between characteristics of medical students’ co-regulatory networks, perceived learning opportunities, and self-regulated learning.

**Methods:**

The authors surveyed 403 undergraduate medical students during their clinical clerkships (response rate 65.5%). Using multiple regression analysis, structural equation modelling techniques, and analysis of variance, the authors explored relationships between co-regulatory network characteristics (network size, network diversity, and interaction frequency), students’ perceptions of learning opportunities in the workplace setting, and self-reported self-regulated learning.

**Results:**

Across all clerkships, data showed positive relationships between tie strength and self-regulated learning (β = 0.095, *p* < 0.05) and between network size and tie strength (β = 0.530,* p* < 0.001), and a negative relationship between network diversity and tie strength (β = −0.474,* p* < 0.001). Students’ perceptions of learning opportunities showed positive relationships with both self-regulated learning (β = 0.295, *p* < 0.001) and co-regulatory network size (β = 0.134, *p* < 0.01). Characteristics of clerkship contexts influenced both co-regulatory network characteristics (size and tie strength) and relationships between network characteristics, self-regulated learning, and students’ perceptions of learning opportunities.

**Discussion:**

The present study reinforces the importance of co-regulatory networks for medical students’ self-regulated learning during clinical clerkships. Findings imply that supporting development of strong networks aimed at frequent co-regulatory interactions may enhance medical students’ self-regulated learning in challenging clinical learning environments. Social network approaches offer promising ways of further understanding and conceptualising self- and co-regulated learning in clinical workplaces.

**Supplementary Information:**

The online version of this article (10.1007/s40037-021-00664-x) contains supplementary material, which is available to authorized users.

## Introduction

Clinical clerkships are challenging learning environments in which medical students often struggle to self-regulate their learning [1]. Self-regulated learning (SRL) involves formulating learning goals, planning, implementing and adjusting strategies to achieve goals while monitoring progression, followed by self-reflection and formulation of new learning goals [2]. Research increasingly acknowledges that personal, social, and contextual attributes interact to influence medical students’ SRL in clinical workplace settings [3–6]. SRL not only depends on an individual student’s abilities and capacities, but also on available or perceived learning opportunities and opportunities to interact with others [7]. The context-dependency and social embeddedness add to the complexity of SRL in the dynamic and unpredictable learning environments of healthcare settings. The notion that social interactions influence students’ regulation of learning is captured in the concept of co-regulated learning (CRL). In CRL, students jointly regulate their learning processes together with peers, residents, or others present in the clinical learning environment [8–10]. For example, conversations with supervising residents or physicians may help students to formulate realistic learning goals, develop and implement learning strategies, or reflect on professional competence development [11].

In clinical clerkships, SRL and CRL are thus inextricably linked, as SRL largely comes about through interactions in students’ social networks. As interactions within these networks specifically focus on, influence, and contribute to students’ SRL, they can be conceptualized as “co-regulatory networks”. Given the importance of enhancing medical students’ SRL in clinical settings, a better understanding of CRL and how co-regulatory networks impact medical students’ SRL is essential. In alignment with shifting conceptualizations of SRL as socially embedded learning activities, adopting a social network approach seems eminently suitable to explore relationships between medical students’ co-regulatory networks and their self-regulatory learning behaviours [12].

Social networks are structures consisting of actors (individuals) and links between individuals (i.e., ties) that capture various features, such as communication patterns as well as the frequency and content of the communication [13]. Networks are often described in terms of their characteristics. Quantitative approaches to social network analysis consider size, diversity, and tie strength key characteristics of social networks.[14]. *Network size* indicates the number of individuals with whom a person interacts within the network [14]. *Strength of ties* indicates the frequency or duration of interactions between individuals in a network [15]. *Network diversity *indicates the degree of variation among individuals within a network (e.g., differences in age, gender, or job level) [16].

Previous research findings suggest that how students interact with others in their networks and how they position themselves within networks influences how, what, and from whom they learn. For example, exchanging relevant information through informal social interactions within networks has been shown to be positively related to medical students’ learning outcomes [17], and students’ network sizes are positively associated with academic performance [18]. Strong ties within networks appear to be particularly important when engaging in complex tasks, whereas weak ties seem to be more important for receiving unique information [19]. Research within organizational contexts furthermore indicates that high performing individuals tend to have highly diverse networks [20]. Research into the relationship between SRL and social networks in virtual learning environments suggests that it is the ‘good’ self-regulators who position themselves in the centre of a network from the very start of engagement in the new environment, creating connections with many others in their network [21].

These studies, conducted outside of clinical workplace settings, highlight the importance of focusing on networks for understanding relationships between networks, SRL, and learning. Although it is widely acknowledged that regulation of learning in clinical clerkships is socially grounded, research explicitly focusing on co-regulatory networks has yet to gain momentum. As SRL research in clinical settings has been largely conducted using, for example, interviews and focus groups, we aim to expand existing knowledge by examining relationships between SRL and students’ co-regulatory networks through use of a quantitative social network approach that enables us to explore the structure of co-regulatory networks in a systematic way. More specifically, this study aims to explore and describe relationships between characteristics of medical students’ co-regulatory networks in clinical settings (network size, network diversity, tie strength), students’ perceptions of learning opportunities, and their self-reported self-regulated learning.

## Method

### Methodology

Our purpose with this cross-sectional questionnaire study is exploratory and descriptive. We were particularly interested in exploring networks of relations and interactions surrounding individual students rather than focusing on all relationships within the clinical learning environment as a whole. We administered a questionnaire to explore various aspects of students’ SRL, students’ perceptions of the workplace learning context, and their co-regulatory networks during clinical clerkships. To collect our data, we drew on previous research indicating that self-reports are often used to study SRL [22, 23] and social networks [12, 14, 17].

### Setting

We conducted this study in the undergraduate master’s in medicine programme at Maastricht University, the Netherlands. The programme is designed according to principles of competency-based medical education [24]. Students complete three years of clinical clerkships in an academic hospital and affiliated teaching hospitals (five regular clerkships, two electives, one healthcare participation clerkship [HELP], and one scientific research participation clerkship). Students rotate through clerkships in a fixed order, starting with internal medicine or surgery and finishing with HELP. Clerkships last between 8 and 18 weeks and predominantly consist of workplace learning; mandatory educational meetings are scheduled at regular intervals. The programme supports SRL through an e‑portfolio, mentors, and assigned workplace supervisors. Students formulate learning goals and plans, discussing these with their mentor and the assigned workplace supervisor at the start of every clerkship [25].

### Participants and data collection

Students were eligible if they, at the time of our study, were enrolled in one of the following clerkships: internal medicine, surgery, neurosciences, mother and child, family and social medicine, or HELP (*N* = 615). Between November 2019 and February 2020, DB recruited students during 41 educational meetings that were spread across all clerkships. After a short explanation of the study, DB handed out QR codes and URL links that provided access to the questionnaire. The Ethical Review Board of the Dutch Society for Medical Education approved this study (ref. 2019.2.3).

### Instrument

We administered a two-part questionnaire. The first part focused on SRL behaviours and students’ perceptions of the extent to which the workplace learning context entailed opportunities for learning and SRL; the second part focused on students’ co-regulatory networks. Acknowledging the notion that networks and student behaviours within particular networks may vary across contexts, we requested participants to keep in mind their current clerkship when completing the questionnaire. Prior to administration, the complete questionnaire was pilot tested with 10 respondents. After the pilot, we made minor adjustments to several items to improve comprehensibility. Additionally, we based response options for the co-regulatory network questionnaire on pilot respondents’ input [26], which provided initial estimates of network sizes and interaction frequencies as well as relevant others with whom students in clerkships interact.

#### Self-regulated learning at work questionnaire

We used an adapted version of the Self-Regulated Learning at Work Questionnaire (SRLW-Q), which was constructed and validated in workplace settings [7]. We included the subscales appropriate for our context, i.e., the forethought, performance, and self-reflection scales, henceforward referred to as the SRL scale, and the workplace learning context scale, henceforward referred to as the WLC scale, as a measure of perceived learning opportunities in a particular clerkship setting. Using principles of collaborative and iterative translation [27], we translated and adapted both scales to the study setting. DB translated the items and, in collaboration with SRL experts, clinical workplace learning experts, and questionnaire design experts, iteratively refined items until the expert panel perceived the match with clinical workplaces to be appropriate (see the Appendix in the Electronic Supplementary Material [ESM] for the complete questionnaire).

#### Co-regulatory network questionnaire

The second part of the questionnaire focused on students’ co-regulatory networks. Participants indicated whom they engaged with to discuss SRL activities as described in the SRL scale. Participants could select one or more groups out of eight options (e.g., peers, residents, physicians). After identifying relevant relationships, participants indicated from a fixed number of options how many individuals within each selected group they engaged with (providing measures of network size and diversity) and the interaction frequency with members of that particular group (providing a measure of tie strength). Of note: based on the pilot we learned that the cognitive load required to complete the original questionnaire was high. Thus, we decided to measure interaction frequency for groups as a whole rather than for each separate individual in the student’s network.

### Network measures and data analysis

We calculated the variable ‘network size’ by counting the total number of individuals in a student’s network, and the variable ‘network diversity’ by counting the number of different groups present within a student’s network. We calculated the variable ‘tie strength’ by adding interaction frequencies with groups within a student’s network and dividing this sum by the number of groups present in the network, providing a measure of mean tie strength. To provide a measure of tie strength for each clerkship as well as across clerkships, mean tie strengths were calculated by averaging means across the various groups. We computed the internal consistency for the SRL and WLC scales. Structural equation modelling (SEM) was used to investigate relationships between the variables included in this study. We first conducted several multiple regression analyses to explore relationships between network size, network diversity, tie strength, WLC, and SRL. Results from these regressions provided input for constructing the investigated model. We checked the distributions of the variables in the SEMs for normality and correlations and measured the quality of fit for the SEMs by comparative fit index (CFI), Tucker-Lewis index (TLI), and root mean square error of approximation (RSMEA). We conducted a multiple group analysis of the relationships, in which we included students’ current clerkships. Since multiple group-invariance analysis on clerkship revealed differences between clinical clerkships regarding relationships within the model, we conducted analysis of variance (ANOVA) to compare co-regulatory network characteristics between clerkships. We performed analyses using R 3.6.3, and R‑package Lavaan 0.6–5 (R Foundation for Statistical Computing, Vienna, Austria).

## Results

Of the 615 students invited to participate, 403 (65.5%) students completed the questionnaire. Of those who completed the questionnaire, 145 (36%) were first-year students, 142 (35%) were second-year students, and 116 (29%) were third-year students. The sample consisted of 284 women (70.5%) and 117 men, which is representative of the student population in the programme (69% women). Table S1, found in the Electronic Supplementary Material (ESM), presents descriptive statistics for network size, tie strength, network diversity, and the SRL and WLC scales for each clerkship and across clerkships. The Cronbach’s α of the SRL and WLC scales was 0.893 and 0.693, respectively.

**Fig. 1:**
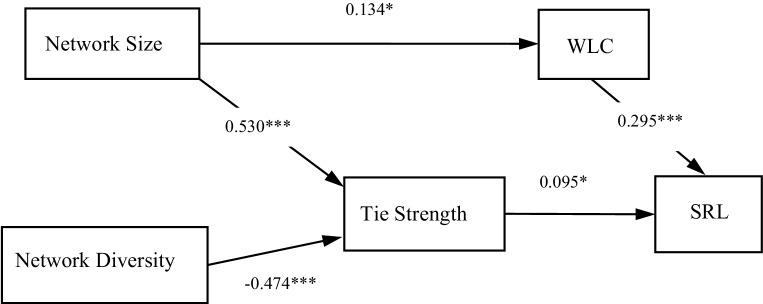
Model of relationships among co-regulatory network characteristics, WLC and SRL. The Structural Equation Model is informed by multiple regressions for the variables network size, network diversity, tie strength, workplace learning context scale (WLC), and self-regulated learning scale (SRL). We present the standardized coefficients. (* = *p* < 0.05, *** = *p* < 0.001)

Fig. 1 presents the structural equation model and relationships between students’ co-regulatory network characteristics, SRL, and WLC. First, we analysed relationships between variables across all clerkships. Fit parameters for the analysis across clerkships were good (CFI = 0.923; TLI = 0.826; RMSEA = 0.065). Fit parameters for the multiple group analysis were slightly better (CFI = 0.985; TLI = 0.967 RMSEA = 0.029). The multiple group analysis indicated configural variance of the model between clinical clerkships, which shows that students’ current clerkship contexts moderated relationships in the model.

Tab. 1 presents results from analysing relationships within the SEM, both across (overall) and for each clerkship. When analysing relationships across clerkships, all direct relationships within the model were significant. We found a positive relationship between tie strength within co-regulatory networks and SRL (β = 0.095, *p* < 0.05), indicating that an increase in interaction frequency is associated with an increase in self-reported SRL. Although we found no significant relationships between network size and SRL, nor between network diversity and SRL, we found that network size related positively to tie strength (β = 0.530, *p* < 0.001), whereas network diversity related negatively to tie strength (β = −0.474, *p* < 0.001). We found positive relationships between WLC and SRL (β = 0.295, *p* < 0.001) and between network size and WLC (β = 0.134, *p* ≤ 0.05). As Tab. 1 shows, multiple group analysis revealed differences between clinical clerkships regarding relationships within the model. This moderation is evidenced by positive relationships between SRL and tie strength in some but negative relationships in other clerkships. Relationships between WLC and SRL were more robust, evidenced by consistent positive relationships.

**Table 1:** Structural equation model results (*N* = 403)

	Overall (*N* = 403)	IM (*N* = 75)	SC (*N* = 70)	NS (*N* = 81)	MC (*N* = 56)	FSM (*N* = 83)	HELP (*N* = 38)
TS → SRL	0.045* β = 0.095	0.009 β = 0.018	−0.053 β = −0.110	0.038 β = 0.080	0.162* β = 0.337	0.194*** β = 0.403	−0.009 β = −0.019
ND → TS	−0.234*** β = −0.474	−0.060 β = −0.122	−0.471*** β = −0.954	−0.066 β = −0.133	−0.221 β = −0.448	−0.045 β = −0.092	−0.155 β = −0.314
NS → TS	0.113*** β = 0.530	0.014 β = 0.066	0.196*** β = 0.923	0.065 β = 0.304	0.102 β = 0.479	0.042 β = 0.196	0.101* β = 0.476
WLC → SRL	0.241*** β = 0.295	0.381*** β = 0.467	0.373*** β = 0.457	0.212* β = 0.260	0.192* β = 0.235	0.257* β = 0.315	0.314 β = 0.385
Size → WLC	0.017** β = 0.134	0.030 β = 0.241	0.001 β = 0.010	0.005 β = 0.039	0.029 β = 0.232	−0.003 β = −0.028	0.014 β = 0.113

Structural equation model results across clerkships (overall) and within clerkships

Abbreviations/Explanations: *IM* Internal Medicine, *SC* Surgical Clerkship, *NS* Neurosciences, *MC* Mother and Child, *FSM* Family and Social Medicine, *HELP* Healthcare Participation Clerkship. We mention unstandardized estimates and standardized estimates (*β*) for relationships within the structural equation model (*ND* network diversity, *NS* Network Size, *TS* Tie Strength, *SRL* Self-Regulated Learning Scale, *WLC* Workplace Learning Scale)

*p < 0.05, **p < 0.01, ***p < 0.001

Figure S1 (in ESM) provides an overview of students’ co-regulatory network characteristics in six different clerkships. For each clerkship, it presents students’ co-regulatory networks, depicting whom students include in their networks, the number of individuals within each group, and the interaction frequency with each group. Although co-workers, and particularly peers and residents, fulfil a prominent role in students’ co-regulatory networks, Fig. S1 shows that, throughout the programme, students seem to also engage friends and family in efforts to regulate their learning. Figure S1 furthermore shows that mentors are among the least frequently engaged across all clerkships as reflected by their relatively low tie strengths. Table S2 (in ESM) presents ANOVA results, comparing co-regulatory network characteristics between clerkships. We found significant differences in network size and tie strength, but not in network diversity. As shown, tie strength was strongest in surgery clerkships (*M* = 3.57) and weakest in HELP clerkships (*M* = 2.64). Co-regulatory network size was largest in surgery clerkships (*M* = 9.23) and smallest in family and social medicine clerkships (*M* = 7.29).

## Discussion

This exploratory study aimed to describe relationships between students’ co-regulatory network characteristics (size, diversity, tie strength), students’ perceptions of learning opportunities in clinical learning contexts, and self-reported SRL. We found positive and significant relationships between tie strength and SRL and between perceived learning opportunities and SRL. The clerkship context influenced both co-regulatory network characteristics as well as relationships between co-regulatory network characteristics, SRL, and perceived learning opportunities.

Overall, our findings confirm the importance of relationships and interactions in co-regulatory networks for medical students’ SRL in clinical settings. By elucidating the scope of co-regulation in clinical workplaces, this study builds on, contributes to, and reinforces changing conceptualizations of SRL as socially embedded learning activities [11, 23]. Medical education research into SRL has shown that formulating learning goals in clinical settings requires learners and engaged supervisors to interact and negotiate for goals to be realistic and achievable [28–30], as well as to monitor performance [31]. We expanded this research by adopting a social network approach to CRL and SRL, allowing our data to show the extent to which students engage in CRL, and more specifically with whom, with how many others, and how often students co-regulate their learning during clerkships. We found that strong ties within co-regulatory networks in particular seem to benefit students’ self-reported SRL. This finding seems to corroborate research that highlights the importance of social interactions for self-regulation in clinical contexts [5, 6, 11, 30–33], but refines this notion by suggesting that co-regulatory interaction frequency might be an important characteristic requiring further investigation. Our findings also suggest that being embedded in large co-regulatory networks might facilitate interaction frequency, further enhancing medical students’ SRL.

Our results furthermore suggest that students’ ability to recognize learning opportunities within a particular context is essential for SRL, thus confirming previous research findings highlighting students’ ability to align learning opportunities with learning plans [34]. Interactions within co-regulatory networks may support such recognition processes, especially if they are targeted at helping students to become aware of learning opportunities. Our findings regarding differences between clerkships in co-regulatory network characteristics and their relationships with SRL further highlight the context-dependency of SRL and CRL in clinical learning environments. These differences may reflect clerkships’ context-specific features affecting co-regulatory network sizes, such as the number of supervisory staff present, with fewer staff at general practices compared to surgical departments at hospitals, for example. Similarly, one possible explanation for the variations in tie strength is that they may reflect differences in availability and approachability of supervisors in different clerkship settings [35], influencing students’ tendency to include multiple others in their co-regulatory network to support their learning and SRL. Self-evidently, there may be multiple explanations for differences between clerkships in co-regulatory network characteristics and their relationships with SRL, such as differences inherent to the clerkship specialty, instructional support, or the structure of the clerkship itself.

An interesting nuance is our finding that tie strength was weakest in HELP clerkships. Given these clerkships are at the end of the master’s programme, preceding transition towards residency training, lower co-regulatory interaction frequency may reflect students’ pull toward acting autonomously and independently [36]. Autonomy and independence are core values within the culture of medicine, and students competing for positions in residency training may feel pressured to conform to perceived or explicit expectations to act independently [37, 38]. The prevailing culture within medical education regarding progressive independence may thus inhibit students’ willingness to engage others in their learning, even if they endorse the value of CRL. Additionally, students’ CRL goals may change over time, potentially affecting interaction frequency as well. A recent study, for example, showed that experienced students, compared to novices, focused less on task-specific aspects of medical practice and more on professional identity development when engaging others in their learning [11]. Students’ increasing confidence or competence in task performance and increasingly urgent considerations about the kind of physician they want to become may thus result in differently oriented, yet less frequent CRL.

### Practical implications

Clinical contexts should provide students opportunities to build networks in which frequent co-regulatory interaction is stimulated. One approach is to stimulate partnerships among students, as well as between students and staff, for example, in prolonged clerkships, in which students are provided opportunities to build longitudinal relationships with others. Students and supervisors may then be encouraged and facilitated to establish learning needs and goals collaboratively, and to frequently interact regarding how to achieve shared goals in healthcare as well as in student’s competence development [39].

Aligned with our finding that co-regulatory network size contributes to tie strength, mentors and supervisors can help students develop large co-regulatory networks that provide opportunities for frequent co-regulatory interaction. We recommend that faculty development programmes pay attention to development of relevant skills to coach students in development of CRL skills and network building, as well as help students recognize and use available learning opportunities in various workplace settings. Additionally, rather than focusing students’ SRL training on individual skills (e.g., goal setting, self-assessment, and reflection), medical education programmes might focus attention on development of skills that enable students to engage in CRL, such as feedback seeking and engaging others in learning conversations. This should include activities to make students aware of the benefits of co-regulatory networks and foster their ability to act on these benefits intentionally. Research in teacher education, for example, shows that network training sessions can be effective in developing quality networks [40]. These findings might provide a framework for designing training programmes targeting medical students’ network building skills.

### Limitations and future directions

First, we captured complex regulatory constructs using a questionnaire, reducing reality to response options within the questionnaire. Second, we based our conclusions on students’ self-reports about complex SRL and CRL behaviour. Students may have varied in understanding questionnaire items or might not have been able to assess themselves or their networks accurately. However, self-reports are commonly used to study both SRL [22, 23] and networks [12, 14, 17]. Third, we focused on student-initiated interaction. Overall network size, diversity, and tie strength might have been larger had we included interaction initiated by others in clinical workplaces. Fourth, participants indicated the number of individuals within their co-regulatory networks from a fixed number of options. Therefore, network sizes may be larger than our data reflect. Fifth, we focused on only one characteristic of the individuals present in students’ networks. That is, we only explored to which group they belonged (e.g., whether they belonged to the group of peers or physicians). We attempted to reduce these limitations by rigorous pilot testing of the questionnaire. Notwithstanding these limitations, our study was a first exploratory, quantitative attempt to explore co-regulatory networks in clerkships.

Our findings uncover future research directions to further disentangle SRL and CRL in clinical settings. First, researchers could focus on CRL initiated by persons other than students, to capture the mutuality of CRL in clinical settings. Second, future research might want to consider building on recent trends in network research that point to using mixed method social network analysis, which combines qualitative and quantitative approaches to analyse networks and allows for investigating both structural characteristics of networks and the meaning of interactions [41]. Drawing on mixed method designs allows us to improve our understanding of the full complexity of interactions in co-regulatory networks. Third, future research could also focus on other characteristics of individuals in students’ networks, such as experience (both as a healthcare professional and clinical teacher), gender, and age. Examining multiple characteristics helps create a more detailed understanding of the individuals with whom students engage in the regulation of their learning. A method for generating these data might be the use of predefined recall lists (or rosters) which present names of individuals, asking participants to indicate with whom on the roster they maintain specific relationships for regulation of their learning [12].

## Conclusions

This study highlights and reinforces the social and contextual embeddedness of SRL and CRL. Results provide insight into relationships among co-regulatory network characteristics, SRL, and clinical learning contexts, accentuating the importance of frequent interactions with meaningful others and making students aware of available learning opportunities. With its social network orientation, this study offers methods for operationalizing SRL and CRL in clinical workplaces, thereby paving a way along which medical education research can continue to disentangle social, relational, and contextual factors influencing SRL.

## Supplementary Information

**Table S1**. Descriptive statistics for co-regulatory network characteristics, self-regulated leaning scale, and workplace learning context scale (*N* = 403)

**Table S2**. Means, standard deviations, ANOVA tests comparing network characteristics of six clinical clerkships, (*N* = 403)

**Fig. S1.** Medical students’ co-regulatory networks within six clinical clerkships. The figure presents the mean number of peers, residents, physicians, workplace supervisors (WPS), mentors, nurses, friends, and family members in parentheses (the larger the line endpoint, the larger the number of individuals within that group), and the mean tie strength for each group next to the spokes that connect the student to the various groups (the thicker the spoke lines, the higher the interaction frequency). Mean tie strengths were calculated by averaging means across the various groups both within clerkships and across clerkships

**Appendix**: Instrument
