# Supplementary material for: Relationships between medical students’ co-regulatory network characteristics and self-regulated learning: a social network study
Source: Perspect Med Educ. 2021 Apr 30;11(1):28–35. doi: 10.1007/s40037-021-00664-x (PMC8733107; doi:10.1007/s40037-021-00664-x)
Supplement: Supplementary file 1 — Table S1. Descriptive statistics for co-regulatory network characteristics, self-regulated leaning scale, and workplace learning context scale (N = 403) [file 40037_2021_664_MOESM1_ESM.docx]

| **Table S1** Descriptive statistics for co-regulatory network characteristics, self-regulated leaning scale, and workplace learning context scale (*N*=403) | | | | | | | | | | | |
| --- | --- | --- | --- | --- | --- | --- | --- | --- | --- | --- | --- |
|  | *N* | **Network Size** | | **Network Diversity** | | **Tie Strength** | | **SRL** | | **WLC** | |
|  |  | *M* (1–27) | *SD* | *M* (1–8) | *SD* | *M* (1–5) | *SD* | *M* (1–5) | *SD* | *M* (1–5) | *SD* |
| **IM** | 75 | 8.37 | 3.56 | 4.09 | 1.63 | 3.23 | 0.73 | 3.51 | 0.33 | 3.55 | 0.40 |
| **SC** | 70 | 9.23 | 4.30 | 3.97 | 1.79 | 3.57 | 0.83 | 3.44 | 0.37 | 3.71 | 0.43 |
| **NS** | 81 | 7.59 | 3.62 | 4.28 | 1.57 | 2.91 | 0.76 | 3.48 | 0.41 | 3.29 | 0.48 |
| **MC** | 56 | 7.52 | 4.07 | 4.05 | 1.75 | 3.05 | 0.85 | 3.63 | 0.44 | 3.43 | 0.57 |
| **FSM** | 83 | 7.29 | 3.77 | 4.12 | 1.69 | 3.14 | 0.82 | 3.45 | 0.41 | 3.40 | 0.49 |
| **HELP** | 38 | 8.61 | 3.52 | 4.74 | 1.43 | 2.64 | 0.62 | 3.55 | 0.40 | 3.60 | 0.38 |
| **Total** | 403 | 8.04 | 3.86 | 4.17 | 1.66 | 3.13 | 0.82 | 3.50 | 0.39 | 3.48 | 0.48 |
| Abbreviations/Explanations: Means (*M*), Standard Deviations (*SD*) for network size, network diversity, tie strength, self-regulated learning scale (*SRL*), and workplace learning context scale (*WLC*) within six clinical clerkships (*IM* Internal Medicine, *SC* Surgery Clerkship, *NS* Neurosciences, *MC* Mother and Child, *FSM* Family and Social Medicine, *HELP* Healthcare Participation Clerkship) and for all students (*Total*). Mean tie strengths were calculated by averaging means across the various groups both within clerkships and across clerkships (total) | | | | | | | | | | | |
