# Supplementary material for: Relationships between medical students’ co-regulatory network characteristics and self-regulated learning: a social network study
Source: Perspect Med Educ. 2021 Apr 30;11(1):28–35. doi: 10.1007/s40037-021-00664-x (PMC8733107; doi:10.1007/s40037-021-00664-x)
Supplement: Supplementary file 2 — Table S2. Means, standard deviations, ANOVA tests comparing network characteristics of six clinical clerkships, (N = 403) [file 40037_2021_664_MOESM2_ESM.docx]

| **Table S2** Means, standard deviations, ANOVA tests comparing network characteristics of six clinical clerkships, (*N*=403) | | | | | | | |
| --- | --- | --- | --- | --- | --- | --- | --- |
|  | **Overall** | **IM** | **SC** | **NS** | **MC** | **FSM** | **HELP** |
| **Network Size** | 8.05 (3.86) | 8.37 (3.56) | 9.23 (4.30) | 7.59 (3.62) | 7.52 (4.07) | 7.29 (3.77) | 8.61 (3.52) |
| ANOVA | **F=2.71*** |  | FSM |  |  | SC |  |
| **Tie Strength** | 3.13 (0.82) | 3.23 (0.73) | 3.57 (0.83) | 2.91 (0.76) | 3.05 (0.85) | 3.14 (0.82) | 2.64 (0.62) |
| ANOVA | **F=9.05***** | HELP | NS, MC, FSM, HELP | SC | SC | SC, HELP | IM, SC  FSM |
| **Network Diversity** | 4.17 (1.66) | 4.09 (1.63) | 3.97 (1.79) | 4.28 (1.57) | 4.05 (1.75) | 4.12 (1.69) | 4.74 (1.43) |
| ANOVA | F=1.265 |  |  |  |  |  |  |
| Results from ANOVA tests comparing network size, tie strength and network diversity among clinical clerkships (*IM* Internal Medicine, *SC* Surgical Clerkship, *NS* Neurosciences, *MC* Mother and Child, *FSM* Family and Social Medicine, *HELP* Healthcare Participation Clerkship). We mention means and standard deviations (in parentheses). Mean tie strengths were calculated by averaging means across the various groups both within clerkships and across clerkships. For one-way ANOVA’s we mention the F(5.397) value; for post-hoc tests we mention only those rotations that differ significantly following the pairwise *t*-test  **p<0.05, ***p <0.001)”* | | | | | | | |
