# Supplementary material for: Relationships between medical students’ co-regulatory network characteristics and self-regulated learning: a social network study
Source: Perspect Med Educ. 2021 Apr 30;11(1):28–35. doi: 10.1007/s40037-021-00664-x (PMC8733107; doi:10.1007/s40037-021-00664-x)
Supplement: Supplementary file 3 — Fig. S1. Medical students’ co-regulatory networks within six clinical clerkships. The figure presents the mean number of peers, residents, physicians, workplace supervisors (WPS), mentors, nurses, friends, and family members in parentheses (the larger the line endpoint, the larger the number of individuals within that group), and the mean tie strength for each group next to the spokes that connect the student to the various groups (the thicker the spoke lines, the higher the interaction frequency). Mean tie strengths were calculated by averaging means across the various groups both within clerkships and across clerkships [file 40037_2021_664_MOESM3_ESM.docx]

**Fig. S1** Medical students’ co-regulatory networks within six clinical clerkships. The figure presents the mean number of peers, residents, physicians, workplace supervisors (WPS), mentors, nurses, friends, and family members in parentheses (the larger the line endpoint, the larger the number of individuals within that group), and the mean tie strength for each group next to the spokes that connect the student to the various groups (the thicker the spoke lines, the higher the interaction frequency). Mean tie strengths were calculated by averaging means across the various groups both within clerkships and across clerkships

Peers (2.59)

Residents

(2.00)

Physicians (0.61)

WPS

(0.80)

Mentor

(0.35)

Nurses (0.15)

Friends (1.32)

Family (0.56)

3.67

3.03

1.03

1.49

0.43

0.32

2.05

1.12

Peers (3.43)

Residents

(1.56)

Physicians (0.84)

WPS

(0.54)

Mentor

(0.43)

Nurses (0.31)

Friends (1.30)

Family (0.81)

4.07

2.26

1.70

1.11

0.54

0.64

2.19

1.51

Internal Medicine (*N*=75)

Peers (2.30)

Residents

(1.65)

Physicians (0.77)

WPS

(0.79)

Mentor

(0.36)

Nurses (0.23)

Friends (1.00)

Family (0.49)

3.25

2.86

1.54

1.62

0.38

0.57

1.56

0.86

Neurosciences (*N*=81)

Peers (2.09)

Residents

(1.45)

Physicians (0.77)

WPS

(0.84)

Mentor

(0.50)

Nurses (0.11)

Friends (1.09)

Family (0.68)

3.05

2.38

1.52

1.80

0.55

0.20

1.66

1.18

Mother and Child (*N*=56)

Peers (2.57)

Residents

(1.11)

Physicians (0.58)

WPS

(0.99)

Mentor

(0.37)

Nurses (0.14)

Friends (0.98)

Family (0.55)

3.18

2.12

1.25

2.81

0.43

0.39

1.86

0.99

Family/Social Medicine (*N*=83)

Peers (1.63)

Residents

(2.24)

Physicians (1.21)

WPS

(1.16)

Mentor

(0.42)

Nurses (0.32)

Friends (1.18)

Family (0.45)

2.66

2.61

1.74

2.18

0.42

0.47

1.82

0.71

Healthcare Participation (*N*=38)

Surgical Clerkship (*N*=70)
