# Supplementary material for: Relationships between medical students’ co-regulatory network characteristics and self-regulated learning: a social network study
Source: Perspect Med Educ. 2021 Apr 30;11(1):28–35. doi: 10.1007/s40037-021-00664-x (PMC8733107; doi:10.1007/s40037-021-00664-x)
Supplement: Supplementary file 4 — Appendix: Instrument [file 40037_2021_664_MOESM4_ESM.docx]

**Appendix: Instrument**

**Self-Regulated Learning at Work Questionnaire**

Please indicate the extent to which the following statements describe your behavior. There are no correct or incorrect responses to these questions: please indicate how you typically behave, rather than how you think you should behave.

Scale: 1 = not at all applicable to me, 2 = sometimes applicable to me, 3 = quite applicable to me, 4 = applicable to me, 5 = very applicable to me.

**SRL Forethought scale**

SRL-FT1: I set personal standards for performance in this clerkship.

SRL-FT2: I set long-term goals (for example: monthly) for myself in order to direct my learning activities

SRL-FT3: I set goals to help me plan my learning activities

SRL-FT4: I set realistic deadlines for learning when I have identified a learning need.

SRL-FT5: In this clerkship, I ask myself questions about each task I want or need to learn before I begin

SRL-FT6: I think of several ways to solve a problem and choose the best one

SRL-FT7: When planning my learning, I adapt strategies that have worked in the past

SRL-FT8: I use specific strategies for different things I need to learn in this clerkship

SRL-FT9: I think I will be able to use what I learn in this clerkship in the future

SRL-FT10: It is important for me to learn new things in this clerkship.

SRL-FT11: Learning that I undertake in this clerkship is important to me.

SRL-FT12: I can remain calm when facing difficulties in this clerkship because I can rely on my abilities.

SRL-FT13: When I am confronted with a problem in this clerkship, I can usually find several solutions.

SRL-FT14: Whatever comes my way in this clerkship, I can usually handle it.

SRL-FT15: My past experiences in clerkships have prepared me well for my future as a physician.

SRL-FT16: I meet the goals that I set for myself in this clerkship.

SRL-FT17: I feel prepared for most of the demands during this clerkship.

**SRL Performance scale**

SRL-PF1: I write down a plan to describe how I hope to achieve my learning goals during this clerkship.

SRL-PF2: I ask myself how what I’m learning is related to what I already know.

SRL-PF4: When learning I make notes (for example: diagrams, etc.) to help organize my thoughts

SRL-PF5: I focus on the meaning and significance of new information

SRL-PF6: I organize my time to best accomplish my goals

SRL-PF7: When I’m learning, I try to relate new knowledge I find to what I already know

SRL-PF8: When I’m learning, I bring together information from different sources (for example: information I receive from people and information from literature)

SRL-PF9: I try to apply ideas from my previous clerkships experiences to this clerkship where appropriate

SRL-PF10: During learning I treat the resources I find as a starting point and try to develop my own ideas from them

SRL-PF11: I try to play around with ideas of my own related to what I am learning

SRL-PF12: In this clerkship I think about possible alternative ways to do my tasks

SRL-PF13: When I can’t understand a task, I ask for help.

SRL-PF14: In this clerkship I try to identify whom I can ask for help if I need it

SRL-PF15: When I am unsure about something I look it up

SRL-PF16: I fill in the gaps in my knowledge by getting hold of the appropriate material

SRL-PF17: When faced with a challenge in this clerkship I try to understand the problem as thoroughly as possible

SRL-PF18: I like opportunities to engage in tasks that require me to learn

SRL-PF19: I prefer tasks that arouse my curiosity, even if I need to learn to achieve them.

**SRL Self-Reflection scale**

SRL-SR1: I know how well I have learned once I have finished a task in this clerkship.

SRL-SR2: I ask myself if there were other ways to do things after I finish a task in this clerkship.

SRL-SR3: I think about what I’ve learned after I finish a task in this clerkship.

SRL-SR4: I think about how what I’ve learned in this clerkship fits in to the ‘bigger picture’ at my education.

SRL-SR5: I think about how what I’ve learned relates to the team I am part of in this clerkship.

SRL-SR6: I try to understand how new information I’ve learned impacts my work in this clerkship.

**Workplace Learning Context Scale: WLC**

Please indicate the extent to which this clerkship provides opportunities for learning and development. There are no correct or incorrect responses to these questions.

Scale: 1 = never, 2 = rarely, 3 = sometimes, 4 = often, 5 = very often or always

WLC1: This clerkship requires me to be creative.

WLC2: I can decide for myself which tasks I want to perform during this clerkship.

WLC3: I have opportunities to develop unique skills during this clerkship.

WLC4: I can vary how I do my work during this clerkship.

WLC5: This clerkship requires a high level of skill

WLC6: This clerkship requires me to learn new things

**Social network questionnaire**

Who do you approach to discuss issues such as:

- What do I want to learn?
- How do I intend to learn that?
- How or where do I find suitable learning opportunities?
- How do I check whether I have learned something?

Check all job titles with whom you discuss such matters:

- Residents
- Peers (fellow-students)
- Workplace supervisors
- Nurses
- Physicians
- Mentor
- Family members
- Friends

For each selected group, participants then indicated, from a fixed number of options, the total number of individuals from each group with whom they interacted to discuss issues such as “What do I want to learn?”, “How do I intend to learn that?”, “How or where do I find suitable learning opportunities?”, “How do I check whether I have learned something?”. Provided response options are between parentheses:

- Residents (5)
- Peers (fellow-students) (5)
- Workplace supervisors (4)
- Nurses (3)
- Physicians (3)
- Mentor (1)
- Family members (3)
- Friends (3)

For each selected group, participants then indicated, from a fixed number of options, how often they interacted with individuals from that group to discuss issues such as “What do I want to learn?”, “How do I intend to learn that?”, “How or where do I find suitable learning opportunities?”, “How do I check whether I have learned something?”. Response options were:

1 = approximately bimonthly

2 = approximately monthly

3 = approximately biweekly

4 = approximately weekly

5 = almost daily
